# The Impact of COVID-19 on Anxiety in Chinese University Students

**DOI:** 10.3389/fpsyg.2020.01168

**Published:** 2020-05-22

**Authors:** Chongying Wang, Hong Zhao

**Affiliations:** ^1^Department of Social Psychology, Zhou Enlai School of Government, Nankai University, Tianjin, China; ^2^Department of General Computer, College of Computer Science, Nankai University, Tianjin, China

**Keywords:** anxiety, university students, COVID-19, China, SAS

## Abstract

COVID-19 had become a pandemic raising concerns of widespread panic and increasing anxiety and stress in individuals all over the world (World Health Organization, 2020a). Lots of countries had closed their schools. As the first country to do so, Chinese colleges and universities were making use of different modes of learning, including online-learning based on different platforms to achieve the goal suggested by Ministry of Education in China, “suspending classes without suspending learning,” since middle February. This paper is the first one which aims to investigate the anxiety of Chinese university students after the outbreak of COVID-19 right before the start of new spring term. 3611 university students (female: male = 1.48:1) aged between 18 to 24 from all over China were enrolled to this study from a top university in China. The Self-Rating Anxiety Scale – SAS (Zung, 1971) was used to assess anxiety 2 days before the start of new spring term in middle February. All four-year undergraduate students were included in the study. The mean SAS score was 40.53 (SD = 10.15), significantly higher than the national norm (Mean = 29.78, SD = 10.07, and *p* ≤ .001), and there were still 557 (15.43%, Mean = 58.75, and SD = 8.9) students met the cutoff of 50 and were screened positive. Comparisons among sexes, grades and majors were also conducted. Significant differences were found between all males and all female (*p* ≤ .001), and between all students majoring arts and sciences in the anxiety sample (*n* = 557, *p* = 0.05). The results also showed that the mean SAS scores were not correlated with the regions they came from/lived in. This study concluded that the Chinese university students showed higher anxiety for COVID-19.

## Introduction

COVID-19, the infection caused by a novel coronavirus detected in December 2019 in Wuhan (Hubei province), is now a pandemic announced by World Health Organization, raising concerns of widespread panic and increasing anxiety in individuals (World Health Organization, 2020a, b). This outbreak has also seen entire cities in China effectively placed under mass quarantine since late January 2020. Brooks et al. (2019) reviewed and reported quarantine could bring “post-traumatic stress symptoms, confusion, and anger. Stressors included longer quarantine duration, infection fears, frustration, boredom, inadequate supplies, inadequate information, financial loss, and stigma.” Some researchers also suggested long-lasting effects (Brooks et al., 2019). Besides, previous researches showed that infectious diseases of uncertainty in recent years such as SARS, Ebola, the 2009 and 2010 H1N1 influenza pandemic, Middle East respiratory syndrome and equine influenza all caused negative psychological effects (Bai et al., 2004; Taylor et al., 2008; Wu et al., 2009; Liu et al., 2012; Sprang and Silman, 2013; Rith-Najarian et al., 2019). In situations that are uncertain and evolving such as COVID-19, it is common to feel stressed, anxious, or upset, among other emotional reactions. Medical staff, children, patients with suspected infection, and quarantined family members have been reported under physical and psychological pressure (Chen et al., 2020; Duan and Zhu, 2020; Wang Y. et al., 2020; Xiang et al., 2020).

Depression and anxiety are both common mental disorders with a prevalence of 10–44% in developing countries and depression is the fourth leading cause of morbidity (Azad et al., 2017). University students are at high risk for depression and anxiety symptoms (Zivin et al., 2009; American College Health Association, 2018) and are exposed to multiple stressors unique to this developmental period (Beiter et al., 2015; Drake et al., 2016). Some studies conducted during SARS and H1NI in China have indicated obvious anxiety and stress of university students and suggested coping strategies (Jia et al., 2003; Chen et al., 2004; Li et al., 2011).

In response to the COVID-19 outbreak, the Chinese Government has ordered a nationwide school closure as an emergency measure to prevent spreading of the infection. Ministry of Education of China and Ministry of Industry and Information Technology of China (2020) suggested “suspending classes without suspending learning,” hence there were over 100 million students in China making use of different modes of learning, including online-learning based on different platforms to achieve the goal starting from middle February 2020. Schools can actively promote a health-conscious schedule, good personal hygiene, encourage physical activities, appropriate diet, and good sleep habits, and integrate such health promotion materials into the school curriculum (Brazendale et al., 2017). University campus life and learning have a critical role in the psychological development of students and the home confinement-related issues were hypothesized having psychological impact on university students. However, how would the closure of schools and online-learning affect university students? Will COVID-19 and quarantine increase or decrease their anxiety? Are there any relations between the high numbers of confirmed cases and their increased anxiety? Though previous studies have indicated increased stress and anxiety during epidemic (Jia et al., 2003; Chen et al., 2004; Li et al., 2011), these study samples were all comparatively small (from 316 to 1200 participants). As COVID-19 is now pandemic and there are rapidly increasing cases and mortalities in some countries, such as Italy, Iran, South Korea, Japan, etc., many countries have announced to close schools and start online-learning immediately. However, no research had been found on the impact of COVID-19 on the anxiety of university students. Our current study conducted in February in China may be of some reference for people related and the implementation of the coping strategies and prevention programs in future. It would also help us to establish a baseline data, and better mental health is likely to improve the academic performance of the students.

This paper aims to investigate the anxiety of Chinese university students after the outbreak of COVID-19 right before the start of new spring term. For all the undergraduate students, this is the first time in their lives to start a new term in this way, no face-to-face classroom learning but totally via internet and online platform. Our hypotheses are university students would have higher anxiety than usual for the start of new term and their most concern is new term and COVID-19.

## Materials and Methods

### Participants

A total of 3800 questionnaires were distributed to undergraduate students aged between 18 to 24 in a top multidisciplinary and research-oriented university directly under the jurisdiction of the Ministry of Education in north China during 10 am February 15 to 10 am February 17, 2020. 3611 (female: male = 1.48:1) valid questionnaires were received, and the response rate is 95.03% (see Table 1). All four-year undergraduate students from all the 26 colleges and schools were included in the study. The participants were also classified into two groups according to their majors and degrees: arts and sciences, in which arts include economics, literature, history, philosophy, foreign languages, law, management, Marxism, Chinese languages and culture, business, tourism and finance, and sciences include mathematics, physics, chemistry, life science, environmental science and engineering, medicine, pharmacology, electronic information and optical engineering, material science and engineering, computer science, cyber science, artificial intelligence, software, and statistics and data science. The participants for this study also represented the distribution of the enrolled students from different regions of China in this university, where there was a high ratio of undergraduate students from Tianjin, Hebei, Shandong, Henan, Shanxi and Sichuan province, and there were several from Hong Kong, Macau, and Taiwan regions. There were also 93 students from Hubei province and 42 from Wuhan city, where the majority of cases affected by COVID-19 were identified.

**TABLE 1:** Demographic characteristics of participants.

Grade	Total number	Males	Females	Arts	Sciences
1	1754	689	1065	863	891
2	1087	433	654	567	520
3	654	283	371	279	375
4	116	49	67	37	79
Total	3611	1454	2157	1746	1865

### Procedure

Following the granting of ethical approval from the university to conduct this study, undergraduate students in all grades from one to four were enrolled through online study platforms and groups recently established for the remote learning in new semester to participate in the study during 10 am February 15 to 10 am February 17, 2020 right before the new semester started. Students who agreed in writing to participate were each given an online questionnaire package to complete and return to the researchers. The questionnaire package used in this cross-sectional study consisted of three components: a sociodemographic questionnaire that required each student to provide their sexes, year of study, city or province they were living, major and colleges, or schools; a measure of student anxiety (the Self-Rating Anxiety Scale); an open question about their most concern.

### The Self-Rating Anxiety Scale

The Self-Rating Anxiety Scale – SAS (Zung, 1971) was used to assess anxiety 2 days before the start of new spring term, within 1 month of COVID-19 outbreak in China. The SAS is a 20-item self-report assessment device built to measure anxiety levels, based on scoring in 4 groups of manifestations: cognitive, autonomic, motor, and central nervous system symptoms. A person should indicate how much each statement applies to him or her within a period of one or two weeks prior to taking the test. Each question is scored on a Likert-type scale of 1–4 (based on these replies: “a little of the time,” “some of the time,” “good part of the time,” “most of the time”). Some questions are negatively worded to avoid the problem of set response. Overall assessment is done by total score. Among the 20 items, 5 were reverse scored. The total raw scores range from 20–80 and then needs to be converted to an “Anxiety Index” score which can then be used on this scale below to determine the clinical interpretation of one’s level of anxiety. The validity and reliability of the instrument has been found to be adequate among Chinese participants. According to Chines norm based on the research on 1158 participants, the levels of anxiety were classified as, 25–49 is normal range; 50–59 is mild anxiety levels; 60–69 is moderate anxiety levels; 70 and above is severe anxiety levels.

### Statistical Analysis

The data were organized and analyzed using SPSS 22.0 software. The surveyed population was divided into different groups according to the SAS scoring criteria. Measurement data were expressed as mean and standard deviation (SD). Counting data are expressed by the number of people (%). The analysis of the relationship between sex, major, grade, region, and anxiety initially used the two sample *t*-test. The correlation betwwen SAS scores and confirmed affected cases in different regions were anaylyzed by Pearson’s product-moment correlation analysis, and *p* < 0.05 on double sides was statistically significant.

## Results

### Overall SAS Mean Scores

The mean SAS score was 40.53 (SD = 10.15; see Table 2), below the cutoff of 50 and significantly lower than that (Mean = 41.93, SD = 10.14) during SARS outbreak in 2003 in China (Chen et al., 2004). One sample *t*-test was conducted and *p* ≤ .001. However, the mean SAS score was significantly higher than that of the national norm (Mean = 29.78, SD = 10.07, and *p* ≤ .001), and there were still 557 (15.43%) students identified as anxious of different levels with the maximum score of 100, whom were from different provinces and at different grades with different majors.

**TABLE 2:** SAS mean scores of participants and comparisons.

Comparisons	Mean (SD)	Number	*t*-test	*P* value
This study	40.53 (10.15)	3611	13802470.44	0.00
National norm	29.78 (10.07)	1158		
This study	40.53 (10.15)	3611	–1797531.03	0.00
SARS study	41.93 (10.14)	316		
This study	40.53 (10.15)	3611	500.62	0.00
H1N1 study: controls	35.8 (6.4)	1200		
This study	40.53 (10.15)	3611	266.02	0.00
H1N1 study: quarantine	37.7 (7.9)	1200		
Males	40.28 (10.99)	1454	–29.80	0.00
Females	40.7 (9.55)	2157		
Arts	40.53 (9.82)	1865	0	0.40
Sciences	40.53 (10.46)	1746		
Those anxious in this study	58.75 (8.9)	557	125391.12	0.00
Those anxious during SARS	55.14 (6.58)	316		
Hubei province	40.07 (11.49)	93	–0.35	0.38
All China	40.53 (10.15)	3611		
Wuhan city	40.51 (10.6)	42	–0.02	0.40
Hubei province	40.07 (11.49)	93		
Wuhan city	40.51 (10.6)	42	–0.00	0.40
All China	40.53 (10.15)	3611		

### Group Comparisons

Two-sample *t*-test was conducted and it was found that there were significant differences on SAS scores between males and females (*p* ≤ .001) but not between students of arts and sciences (*p* = 0.4). SAS mean scores of grade one were found significantly different from those of grade two and three (*p* ≤ .001) but not grade four (*p* = 0.15). There was also significant difference between SAS mean scores of grade two and three (*p* ≤ .001). The SAS mean scores of grade four were found not significantly different from any other grades which may be due to comparatively small sample of grade four.

There were around 21.82% of the participants who replied “good part of the time” or “most of the time” for “I feel more nervous and anxious than usual,” about 12.51% of the undergraduate students who chose “good part of the time” or “most of the time” for “I feel afraid for no reason at all” and 20.41% found themselves upset easily or feel panicky for “good part of the time” or “most of the time.” Furthermore, 1192 (33.01%) participants answered “A little of time” or “Some of the time” for “I feel that everything is all right and nothing bad will happen.” 1370 (37.94%) participants chose “A little of time” or “Some of the time” for “I feel calm and can sit still easily.” In addition, there were 734 (20.33%) students felt weak and got tired easily for “good part of the time” or “most of the time.”

### SAS Score Ranges

Table 3 showed the SAS score ranges of participants. There were 56 participants who were severely anxious among whom 32 males and 24 females. Only one male came from Xiangyang city in Hubei province and the rest 55 participants were from other provinces.

**TABLE 3:** SAS score ranges of participants.

SAS		25–49	50–59	60–69	70–100	Total
		
Anxiety	Level	Normal	Mild	Moderate	Severe	
Subtotal participants		3054	358	143	56	3611
Males		1234	123	65	32	1454
Females		1820	235	78	24	2157
Percentage of males in total males (%)		84.87	8.46	4.47	2.20	100
Percentage of females in total females (%)		84.38	10.89	3.62	1.11	100
Percentage of males in total (%)		34.17	3.41	1.80	0.89	40.27
Percentage of females in total (%)		50.40	6.51	2.16	0.66	59.73
Arts subtotal (%)		1444 (82.70)	190 (10.88)	81 (4.64)	31 (1.78)	1746 (100)
Sciences subtotal (%)		1610 (86.33)	168 (9.01)	62 (3.32)	25 (1.34)	1865 (100)
Grade1 subtotal (%)		1510 (86.09)	153 (8.72)	69 (3.93)	22 (1.25)	1754 (100)
Grade2 subtotal (%)		895 (82.34)	129 (11.87)	43 (3.96)	20 (1.84)	1087 (100)
Grade3 subtotal (%)		556 (85.02)	59 (9.02)	27 (4.13)	12 (1.83)	654 (100)
Grade4 subtotal (%)		93 (80.17)	17 (14.66)	4 (3.45)	2 (1.72)	116 (100)

In Table 4, we exmained the 557 students whose SAS scores was above the cutoff and therefore identified as anxious. According to Chen et al. (2004), the mean scores of students identified as anxious during SARS was 55.14 (SD = 6.58). One sample *t*-test showed that significant difference (*p* ≤ .001) was found between the mean SAS scores during SARS and COVID-19 in this study (mean = 58.75, SD = 8.9). We also conducted two sample *t*-test among students of different grades, and no significant difference was found (see Table 2). Significant differences were found between all males and all female (*p* ≤ .001) and between all students majoring arts and sciences (*p* = 0.05) in the anxiety sample.

**TABLE 4:** The 557 participants whose SAS scores are above 50 and therefore identified as anxious.

Grade	Total number		Males		Females		Arts		Sciences	
					
	Subtotal	Mean (SD)	Subtotal	Mean (SD)	Subtotal	Mean (SD)	Subtotal	Mean (SD)	Subtotal	Mean (SD)
1	244	58.52 (7.98)	98	59.78 (8.81)	146	57.67 (7.24)	140	59.39 (8.14)	104	57.34 (7.60)
2	192	58.81 (10.02)	72	62.52 (13.04)	120	56.58 (6.74)	110	58.69 (8.92)	82	58.96 (11.33)
3	98	59.66 (9.16)	38	59.90 (9.94)	60	59.50 (8.63)	45	59.47 (9.63)	53	59.81 (8.75)
4	23	56.79 (6.35)	12	57.71 (5.22)	11	55.80 (7.25)	7	56.96 (7.64)	16	56.72 (5.69)
Total	557	58.75 (8.9)	220	60.59 (10.53)	337	57.55 (7.42)	302	59.09 (8.67)	255	58.34 (9.16)

We also conducted two sample *t*-test for the 93 students were living in Hubei. Their mean SAS score was 40.07 (SD = 11.49), but no significant difference (*p* = 0.4) was found with comparison to that of all participants (mean = 40.53, SD = 10.15). Among them there were 15 participants whose SAS scores are above 50 (mean = 59.78, SD = 11.82). A student from Xiangyang city was found most anxious and his SAS scores are 100, while others were mildly anxious (mean = 56.88, SD = 5.06). Among the 93 students, there were 42 from Wuhan city and their mean SAS score was 40.51 (SD = 10.6), not significantly different from that of the 93 students (*p* = 0.4), or of the whole participants (*p* = 0.4). There was 7 students from Wuhan city has SAS scores about 50 and were all mildly (No. = 4) or moderately (No. = 3) anxious (Max = 68.75).

### The Correlation Analysis of SAS Scores and Confirmed Affected Cases in Each Province

We also conducted the correlation analysis of the SAS scores and the confirmed cases in each region.

1. Between SAS Scores and Confirmed Cases in Each City except Wuhan or Each Province except Hubei Province.

As the confirmed affected cases in Wuhan is far higher than all other cities or provinces, we first deleted the 42 participants from Wuhan and 126 participants whom we did not know which city they were from (for whom we only knew their provinces), then we got 3444 participants from 296 cities. Pearson’s product-moment correlation analysis was conducted and it was found that *t* = −0.03, df = 110, and *p* = 0.97. The alternative hypothesis is that true correlation is not equal to 0 and 95 percent confidence interval is −0.19 to 0.18. We also conducted the correlation analysis between SAS scores and the confirmed cased in each province except Hubei province and got the same result which confirmed that students’ SAS scores has no significant correlation with the confirmed affected cases of COVID-19 in their cities or provinces.

2. Between SAS Scores and Confirmed Affected Cases in Each City except Hubei Provinces.

We then had a close look of all the cities except Hubei province where COVID-19 first outbroke and spread widely. We conducted the Pearson’s product-moment correlation and found that *t* = -0.13, df = 98, and *p* = 0.89. The alternative hypothesis is true correlation is not equal to 0 and 95 percent confidence interval is −0.21 to 0.18. The results indicated that SAS scores has no significant correlation with the confirmed affected cases of COVID-19 in each city outside Hubei province all over China.

3. Between SAS Scores and Confirmed Affected Cases in Each City in Hubei Provinces.

A Pearson’s product-moment correlation analysis was also conducted between the SAS Scores of 93 students from different cities in Hubei province and the confirmed affected cases in these places. The results showed that *t* = 0.33, df = 91, and *p* = 0.75 and the alternative hypothesis is that true correlation is not equal to 0. The 95 percent confidence interval is −0.17 to 0.24. Therefore, no significant was found either.

### Students’ Main Concern

In the current study, we also designed an open question to ask our participants to write down one sentence of their most concern. The majority of the answers are, such as, when the new term will properly start, if their summer holidays will be shortened then, etc. The Cronbach.α is 0.87. The word cloud of answers to this open question showed that the most obvious words were: start of new term, come on, COVID-19 and school. Some other words they also concerned were: class, online learning, China, anxiety, Wuhan, and teacher, etc.

## Discussion

### Overall Anxiety Is Higher Than Usual and the General Population During COVID-19

Any major epidemic outbreak will have negative effects on individuals and society. A study on the public psychological states of 600 people during COVID-19 outbreak showed that their SAS score was 36.92 (SD = 7.33) and 6.33% had anxiety (Wang C. et al., 2020). Our results indicated that university students had higher anxiety than the general population after the outbreak of COVID-19, which showed that the COVID-19 had negative psychological impact on university students on anxiety at least. This confirmed the previous findings and was in accordance with recent articles urging mental health care for people affected by the epidemic. Studies have suggested that public health emergencies can have many psychological effects on college students, which can be expressed as anxiety, fear, and worry, among others (Mei et al., 2011). There was no such national norm of anxiety level (such as SAS score) for Chinese college students but we compared our results with the SAS scores of university students during SARS and H1NI, and concluded that university students have higher anxiety during COVID-19 than SARS (only in anxious group) and H1N1. Besides, Cao et al. (2020) indicated that 24.9% of medical students were afflicted with experienced anxiety because of the COVID-19 outbreak. Compared to our study, higher percentage of medical students had anxiety than the general university students amid COVID-19. In addition, the results showed that the majority (66.99%) of participants were facing different levels of challenges and found difficult to sit still for a longer time, and there were quite a few of them (15.43%) were identified as anxious of different levels. And there were 20.33% of students felt weak and got tired easily. It had been almost a month that the majority of Chinese people were recommended to stay at home to prevent the spread of COVID-19, and our students have kept a slack hand and also lacked proper exercises and social lives. The results showed that quite a few students were suffering stress, fear or uneasy and being affected by the uncertainty of COVID-19 and all these should be taken into account when teachers were delivering online classes. Compared to SARS in 2003 when university students then experienced same quarantines as now, internet was playing a major role nowadays in speedy information spread, open online discussion, feeling expressions, etc. News report and social media also caused complex emotions, such as depression, anxiety, stress, upset, fear, frustration, anger, etc. about the health staff infected and died, daily life difficulties, social unfair and corruption, helplessness of the ordinary people living in Wuhan, etc. According to the feedback from various psychological counseling telephone hotlines set up for COVID-19 around China, anxiety was one of the main characteristics of callers. Studies have confirmed that individuals who have experienced public health emergencies still have varying degrees of stress disorders, even after the event is over, or they have been cured and discharged from hospital (Cheng et al., 2004; National Health Commission of China, 2015). University students were at an important developmental age for their values and judges, and could be easily affected by the opinions and views from social media, therefore, their emotions were also vulnerable.

### Group Comparisons

Significant sex differences were found and female students showed more anxiety than male students. This confirmed with previous researches that females were more likely to suffer from anxiety (Azad et al., 2017), such as the prevalence of depression and anxiety in Pakistan is 34% (range 29–66% in women and 10–33% in men (Mirza and Jenkins, 2004). But there was no significant difference between students majoring in arts and sciences. However, students in grade one had lower anxiety level than grade two and three while grade two had higher anxiety than grade three. This could be explained that grade two and three students had more academic burdens. The postpone of new term and online-learning, etc. caused by COVID-19 would have more effect on their lives and plan, such as the cancelation of GRE, TOEFL, IELTS in February and March will affect their applications for abroad studies in near future, etc. Especially for students in grade two, they just started the professional curriculum in the second year and the scores for each course are more important than in grade one as the scores would be evaluated if they could be recommended for postgraduate students without examinations in the following year. However, compared to students in grade two, grade three was a more stable year and students became more mature and would have better ability or experience to handle fluctuant emotions. This is why students in grade three had lower anxiety than grade two.

### No Correlation of SAS Scores and Confirmed Affected Cases

In the current study we also investigated if confirmed affected cases of COVID-19 in their cities or provinces had any correlation with students’ increase anxiety, however, no significant correlation was found, even in Wuhan city and Hubei province where had 73% of the confirmed affected cases of whole China (National Health Commission of China, 2020). This showed that university students were not much affected by things happening just around them because the convenience of internet brought a national and international vision for them. Young people nowadays obtain information largely via internet on which information travels. Social media are especially popular among young people, which in turn affect their lives. Young people have a high level of trust in information online and it tends to be the place they look first. Therefore, the confirmed affected cases in each city or province would not affect their anxiety, but rather the direction of public opinion. People had been quarantined for 3 weeks when we conducted this study, and the spread of COVID-19 had been well controlled by Chinese government, therefore, our undergraduate students would no longer worry too much about the confirmed cases in each city considering the whole situation in China was getting better which in contrast to the sharp increase of cases abroad.

### Students’ Main Concern

In line with our hypotheses, their main concerns were “the start of new term,” “come on,” “COVID-19,” and “school”. Even COVID-19 changed their lives and habits in the past month, they were facing the start of new term which would take place online rather than face-to-face. They were anxious but also curious about the new challenges, so “come on” was another hot word to encourage themselves, Wuhan and China. “Come on” was also one of the hottest words during the COVID-19 in China, given government media, social media and donated stuffs used the words very frequently. Some other words they also concerned such as class, online learning, China, anxiety, Wuhan, and teacher, etc. were all hot topics in China too on various social media and closely related to the undergraduate students.

### Limitations of This Study

This study had some limitations. Firstly, our sample was still small, though already much bigger compared to the previous similar studies. Furthermore, our sample was from one top university in north China, hardly representative of all China. Future studies could improve the study design by recruiting more students from different regions of China and also from various universities, such as top ten, top 50, top 100, universities under the jurisdiction of Ministry of Education and those of local government, multidisciplinary universities and academies, etc. Secondly, in this current study we only investigated the anxiety of students, not depression, post-traumatic stress disorder, and other possible mental problems. Thirdly, we did not include the coping strategies and prevention programs in this study which could be explored further in future studies. Additionally, we did not collect the information about if participants were infected or not infected, or with infected family members, and the major source used by students to obtain information about covid19, which could be improved in future studies.

## Conclusion

It was concluded that Chinese undergraduate students during COVID-19 outbreak showed higher anxiety. However, in general the psychological status of university students was fairly good, which laid a good foundation for the new term’s online-learning. A further study after a few weeks of new term comparing their anxiety was also suggested. Furthermore, as this is the first study on the impact of COVID-19 on the anxiety of undergraduate students, this data could also be used as baseline to further explore the causes of the higher anxiety and to take measure to reduce the anxiety in our students.

## Data Availability Statement

All datasets generated for this study are included in the article/supplementary material.

## Ethics Statement

The studies involving human participants were reviewed and approved by the Nankai University Ethics Committee and were in accordance with the 1964 Helsinki Declaration and its later amendments or comparable ethical standards. The participants provided their written informed consent to participate in this study.

## Author Contributions

CW and HZ co-designed the study while HZ conducted the study and analyzed the data. CW interpreted the data, wrote, and revised the manuscript.

## Conflict of Interest

The authors declare that the research was conducted in the absence of any commercial or financial relationships that could be construed as a potential conflict of interest.
